# Teachers’ networked professional learning with MOOCs

**DOI:** 10.1371/journal.pone.0235170

**Published:** 2020-07-02

**Authors:** Bodong Chen, Yizhou Fan, Guogang Zhang, Min Liu, Qiong Wang

**Affiliations:** 1 Department of Curriculum and Instruction, University of Minnesota–Twin Cities, Twin Cities, Minneapolis, United states of America; 2 X-Learning Center, Graduate School of Education, Peking University, Beijing, China; 3 School of Informatics, The University of Edinburgh, Edinburgh, United Kingdom; 4 Department of Distance Education, Beijing Institute of Technology, Beijing, China; University of South Australia, AUSTRALIA

## Abstract

Massive Open Online Courses (MOOCs) are used to support professional learning at scale in many countries. The present study examined a MOOC named Flipped Classrooms that was specially designed for in-service teachers in China. This MOOC was offered for seven consecutive iterations across three years and allowed teachers to re-take this course in connection with their teaching practice. Overall, 16% of all 105,370 learners enrolled in at least two iterations of the MOOC. To understand their learning motivations, their learning engagement within the MOOC, and the connections they forged between the MOOC and their teaching, we conducted a mixed-methods study using multiple data sources including course registration records, course entry surveys, learning performance data, click logs, and semi-structured interviews. Results indicated that teacher-learners re-took the MOOC for various reasons such as refreshing domain understanding, improving grades, and addressing practical problems. Click log analysis found MOOC re-takers with different performance trajectories demonstrated distinct learning patterns across iterations. Qualitative analysis of the interview data revealed additional insights into learning within the MOOC and connections forged by the re-takers between the MOOC and their teaching practice. This study contributes fresh insights into the MOOC literature by investigating MOOC re-takers and sheds light on the promise of using MOOC to support networked professional learning. Implications for future MOOCs and teacher learning opportunities are discussed.

## Introduction

Equitable access to quality teacher professional development (PD) remains a significant challenge in many countries. In China, teacher PD is typically arranged and delivered by local education bureaus in the form of topical lectures offered by invited experts. For in-service teachers from under-served rural areas, such lectures are the only source of PD. While these one-shot lectures could raise awareness of important issues among teachers, the lecture format is found to fall short in achieving real-world changes in educational practice [1]. In contrast, in-service teachers from well-resourced regions of China, and elsewhere, are more likely to be exposed to hands-on PD experiences that are connected to classroom practice and conducive to professional growth [2–4]. To catalyze educational changes in historically under-served regions, it becomes imperative to seek innovative means to provide equitable, quality, and scalable professional learning opportunities to teachers.

This paper provides a glimpse into a nationally concerted effort to develop Massive Open Online Courses (MOOCs) to broaden access to teacher PD in China. MOOCs, which emerged to become a global buzzword in early 2010s [5], are recognized as a potential contributor to teacher PD [6]. A growing number of MOOC providers such as Coursera and edX are partnering with universities to offer teacher PD courses on a wide range of topics such as student engagement, inquiry learning, and classroom research. In China, a nationwide teacher MOOC program was initiated in 2014, and had delivered nearly 50 MOOCs and served more than 3 million in-service and pre-service teachers by 2017 [7]. With concerted support from teacher educators, researchers, and teacher online communities [8], these MOOCs could potentially go beyond one-shot PD lectures and empower teachers to develop educational innovations in local schools. In particular, the flexibility and cost-effectiveness offered by MOOCs hold promise for augmenting traditional forms of teacher PD and alleviating the barriers to quality PD facing teachers from under-served regions [9].

This paper is purposefully focused on one unique sub-population of MOOC learners: *re-takers* who take a same MOOC for multiple times. The significance of investigating the sub-population of re-takers is twofold. First, in the current MOOC literature, studies on re-takers or returning learners are rare except for a few examples [10, 11]. Second, this course re-taking phenomenon found in teacher PD MOOCs may allude to teachers’ acute needs for professional learning that are not met by traditional PD models. In contrast with regular one-time learners, or *one-timers*, the re-takers elect to return to a MOOC for potentially important reasons. Understanding factors that lead them to retake a MOOC is particularly important for the context of teacher professional learning. Compared to another sub-population of learners who avidly take multiple MOOCs [12], these re-takers may have stronger professional needs that drive them to re-take a single MOOC several times. Examining their learning engagement would uncover fresh insights into their motivations and learning journeys, and shed light on the design of future MOOCs and alternative teacher PD pathways. Motivated by these needs, the overarching objective of this study was to provide a rich description of MOOC re-takers and their engagement within and beyond a MOOC. By doing so, this study illuminates the prospect of using MOOCs to support professional learning of in-service teachers in China and elsewhere. For the remainder of this paper, we first review pertinent literature on MOOCs and teacher professional learning. We then introduce the research context and methodology, followed by key findings and general discussion of practical implications.

## Related work

### MOOCs and their development in China

Following the naming of 2012 as “the year of MOOCs,” MOOCs quickly became an international buzzword. Given the appeal of MOOCs for broadening access to education, MOOCs have received great attention in emerging economies. In China, the first MOOC partnership was formed in Spring 2013 between edX and two renowned universities—Peking University and Tsinghua University. Fudan University and Shanghai Jiaotong University, two other top universities based in Shanghai, quickly followed suit, joining Coursera months later. In May 2014, China’s native University MOOC platform was officially launched, spurring a new wave of MOOC experiments involving a broader range of universities [13]. As of late 2017, 14 native MOOCs providers were present in mainland China [14], enabling more than 200 higher education institutions to offer more than 3,000 MOOCs.

In 2016, a concerted effort emerged in China to create a cluster of MOOCs for the professional learning of in-service teachers (see http://tmooc.icourses.cn/). As a scalable and cost-effective alternative, MOOCs are used in different countries to address various challenges related to teacher PD. Given traditional teacher PD programs are falling short in meeting the diverse and emergent needs of teachers [15, 16], teacher educators begin to seek alternatives such as MOOCs to support teacher professional learning. Recent work by Chinese scholars has started to investigate whether MOOCs could become a viable alternative for fulfilling teachers’ acute need for professional learning [16, 17]. Despite these advancements, to what extent and in which ways these MOOCs are supporting teacher professional learning remain largely unknown.

### Learning *in* and *with* MOOCs

Due to the diversity of MOOCs and the learning experiences supported by MOOCs, MOOC research is necessarily multifaceted. Scholars have attempted to trace the development of two MOOC strands (i.e., cMOOC and xMOOC) [18], identify salient research themes [19, 20], and analyze the interdisciplinary nature of MOOCs research [21, 22]. Based on extensive interdisciplinary collaboration, prior research on MOOCs has covered a range of topics such as societal and institutional factors, forecasting and prediction, online discussion forums, self-regulated learning, and peer assessment [22].

The increasingly rich landscape of learning [23] motivates MOOC researchers to not only examine learning *in* a MOOC but also new learning opportunities facilitated by—but external to—the MOOC. In other words, more research is needed to look beyond what happens within MOOCs and examine learning *with* MOOCs in a broader societal sphere.

On the one hand, current research on learning *in* MOOCs is primarily concerned with learner engagement, achievement, and activities [24]. This line of research is linked to public discourse around MOOCs about course completion and learner performance. In response to a prevalent criticism of MOOCs’ low completion rates [25], MOOC researchers argue that the notions of course completion and retention need to be re-conceptualized in the MOOC context [26]. In contrast with a typical college class, MOOC learners are more diverse and could include “active participants” who are actively engaged with all aspects of the course, “passive viewers” who do not complete quizzes or interactive activities, “samplers” who only engage with particular modules, and “curious learners” who never access the MOOC after signing up [27–30]. Typologies of MOOC learners as such highlight an inconsonance between traditional conceptualizations of formal learning and learning in MOOCs and therefore motivate a broader look at learning ecologies that encapsulate MOOC learning [31].

Learning *with* MOOCs takes a stance that a MOOC by itself is insufficient in supporting one’s learning since learning takes place in broader spatial, temporal, and social arenas. For instance, many MOOC learners tend to interact on social media during and after the course period [21]; some form co-located study groups to watch and study MOOC videos together [32]. Recognizing the richness of learning with MOOCs, innovative course designs, such as the “dual-layer MOOCs” [33], are proposed to provide students with multiple learning pathways that integrate varied amounts of in-course and out-of-course learning. Such research and development efforts recognize broader spheres of learning and demonstrate ways to support rich learning *with* MOOCs [23].

### Networked professional learning with MOOCs

The significance of learning *with* MOOCs is contextualized by the need for lifelong and life-wide learning [34] in knowledge societies [35]. Contemporary views of education problematize the factory model of education and seek a deeper fusion between learning and work [23, 36]. As a result, learning has necessarily become more ubiquitous, pervasive, and perpetual. It is especially the case for working professionals who face emerging needs to upskill in order to meet evolving job requirements.

The notion of *networked learning* is aptly aligned with these changes and demands. As one genre of technology-mediated learning, networked learning emphasizes the development and maintenance of a web of social relations between people and between people and resources [37, 38]. In the area of professional development, networked learning is especially relevant because of its emphasis on connections. Networked learning does not demand professionals to time-out from work to learn. Instead, it harnesses connections of professionals to boost their learning, develop new connections as they learn, and hence empower future learning. This type of *networked professional learning* can be facilitated by MOOCs that connect learners to quality resources and learning partners [39, 40]. Treating networks and connections as enablers of learning gives learning professionals more agency to shape their learning experiences in relation to the workplace [37, 41]. By fostering connections that enable further learning, learning professionals are better able to cope with the increasing complexity of modern work and the constant changes with knowledge and practices in the workplace. Below, we explicate three important tenets of networked professional learning that ground this study of professional learning with MOOCs.

#### Fusion of work and learning in a networked society

Problem-solving in knowledge organizations constantly requires an individual, or a team, to learn new things. More than ever learning in professional settings is tied to organizational goals, necessitating the fusion between work and learning. These trends motivate a shift from older models of vocational and continuing education to fresh ideas such as “worker as learner” [42]. Learning at work takes place not only through formal training but also in processes of solving problems, working alongside others, and using shared artifacts [43]. Networked technology systems such as groupware and social media are adopted to catalyse the integration of work and learning and to facilitate social activities that foster professional learning at work [44]. The notion of networked learning is compatible with the goal of integrating learning and problem solving in professional settings [37]. At a fundamental level, networked learning shifts the traditional emphasis of continuing education or professional development on “education” towards “learning” and provides learning professionals more agency over their own learning journeys. Moreover, networked professional learning values “knowledge-in-use” [45] and the notion that professional learning is fundamentally about changing thinking and practice. In the MOOC literature specifically, highly-motivated learners are found to connect MOOC learning with their professional roles and needs [46].

#### Just-in-time learning to meet knowledge needs

Knowledge needs in the process of solving today’s problems are much more fluid and emergent. In this case, predetermined modules or learning components are less useful. The old model of “time-out for learning,” as reflected in traditional teacher PD workshops, is no longer viable for addressing emerging learning needs in today’s professional settings [47]. Instead, learning happens in a just-in-time manner when active problem-solving is ongoing [43]. Such just-in-time learning is largely enabled by the increasing use of information technology and digital networks that keep knowledge workers connected [48]. In recent years, various socio-technical systems have been created to expand learning opportunities in both spatial and temporal dimensions, allowing professionals to learn without leaving the problem at hand. For instance, today’s software programmers are increasingly learning on the fly, with help from social media sites, Q&A forums, and other openly networked spaces [49, 50]. With the mediation of network technologies, professional learning can take place in local and global communities [51, 52]. Networked learning emphasizes connections that enable learning (e.g., following on Twitter or affiliating with a Reddit community) instead of the content knowledge one could acquire via these connections [53, 54]. In such a networked environment, just-in-time learning can happen more naturally, without being detached from practice, and thereby facilitate the fusion of learning and work [53].

#### Diversified attitudes toward credentials and certification of mastery

While professional accreditation remains useful, especially for early career workers [43], it is no long a necessary condition for meaningful professional learning. In formal education, the meaning of learning is often reclaimed by detaching learning from the endless cycle of high-stake tests [55]. In networked professional learning, learning is actively embedded in relations and network structures. Learning happens when networked relations are created and purposefully maintained. This makes it both more difficult to certify learning (given learning is so integral to practice and problem-solving) and less meaningful to provide traditional certification given learning in a networked sense is constantly evolving. Thus, compared to opportunities to advance their knowledge and practice, networked learners tend to “fluidly pursue goals related to their role in society without institutional support or formal training” [56, p. 8]. They are less concerned with certification of mastery that has value but is less valuable compared to solving problems at hand [30]. By the same token, learning professionals who take MOOCs for professional needs are less concerned about course performance than developing useful knowledge and expertise [46]. For many, social capital rather than a record of completion is more valuable in MOOCs and other online learning communities [57, 58].

These tenets of professional networked learning are evident in teacher professional development. Teacher learning is “a process of increasing participation in the practice of teaching, and through this participation, a process of becoming knowledgeable in and about teaching” [59, p. 37]. Networked learning can be an integral part of the teaching practice because “teachers need to see themselves as perpetual learners and be given opportunities to reform their own personal understandings…” [60, p. 13]. Being able to learn in evolving networks of relations conveys a shift with the power dynamics in education that positions teachers’ work as a curriculum of inquiry by itself [61]. In schools, networked learning leads to transformative leadership of teachers that is key for incurring and sustaining changes in schools [62]. On the web, teachers are increasingly using online networks and communities for professional learning [51, 52, 63], opening up new opportunities for just-in-time learning, serendipitous encounters, and peer collaboration among teachers. As MOOCs are developed to support teacher PD at scale, an open question remains: *To what extent and in which ways can MOOCs be mobilized to support networked professional learning of in-service teachers?*

## The present study

In the present study, we tackled this overarching question by investigating a unique sub-population of learners who repeatedly enrolled themselves in a MOOC designed for teacher professional learning. From July 2014 to March 2016, a four-course MOOC series targeting in-service teachers were offered by the X-Learning Center of Peking University in Beijing, China. The most popular MOOC in this series, titled *Flipped Classroom*, was offered seven times during that period, allowing teachers to repeatedly enroll in this MOOC. By focusing on the re-takers, this study aimed to examine the extent to which MOOC re-takers were able to maintain and harness their connection with the MOOC to advance their professional learning goals. Specifically, we asked the following research questions:

*RQ1*: What motivated teachers to re-take the MOOC?
*RQ2*: What did re-takers do within the MOOC?
*RQ3*: What did re-takers do with the MOOC in their professional settings?

To answer these questions, we conducted a mixed-methods study following the *sequential explanatory design* [64]. Specifically, we first analyzed quantitative data using statistical and data mining techniques and then applied qualitative data to help explain and elaborate on quantitative findings [65]. This research design is especially appropriate for MOOC research because while data mining techniques could identify high-level patterns in large MOOC datasets, qualitative analysis could further ground these high-level patterns in thicker depiction of learners’ personal experiences. As of research ethics involved in the study, Peking University as the institution that housed this study provided rigorous research ethics training to its researchers. All researchers involved in this study have participated in such training. The data-mining component of this mixed-methods study used secondary data from the studied MOOC. No explicit consent was obtained because the participants included tens of thousands of MOOC learners and de-identified data were used for analysis. For the qualitative component that involved nine participants in semi-structured interviews, oral consent was obtained before each interview. Throughout the study, participants’ identifiable information has been strictly protected; only pseudonyms are used in research reports.

In the remainder of this section, we explain the research context, participants, data sources, and data analyses.

### Research context and participants

The MOOC aimed to help teachers understand and apply the Flipped Classroom approach [66]. This MOOC comprised six modules, including one course orientation module and five modules on Flipped Classroom concepts, principles, tools, and techniques. Similar to many Coursera and edX MOOCs, this MOOC included video lectures, slides (as PDF files), discussion forums, and quizzes. Learner performance was assessed based on quizzes, homework, and a final exam. Course completion certificates were awarded to learners who achieved at least 60% of all possible points.

Across all seven iterations, this MOOC enrolled a total of 126,044 learners; 10.35% of them received a certificate. Detailed numbers of the MOOC’s enrollment numbers and certification rates can be found in Table 1. Based on a standard entry survey taken by all learners, more than 80% of them were teachers from K-12 and higher education settings.

**Table 1:** MOOC enrollment numbers and completion rates.

Iteration	Duration	Registered Learners #	Certificate #	Certificate %
1	2014/07/01 – 2014/08/10	24,971	5,010	20.06%
2	2014/09/10 – 2014/10/31	29,763	2,425	8.15%
3	2014/12/16 – 2015/01/31	17,924	2,027	11.31%
4	2015/03/18 – 2015/05/07	11,892	1,032	8.68%
5	2015/07/13 – 2015/08/31	19,243	1,579	8.21%
6	2015/10/19 – 2015/12/08	13,264	632	4.76%
7	2016/01/19 – 2016/03/08	8,987	345	3.84%
Total		126,044	13,050	10.35%

#### MOOC re-takers

**Fig 1:**
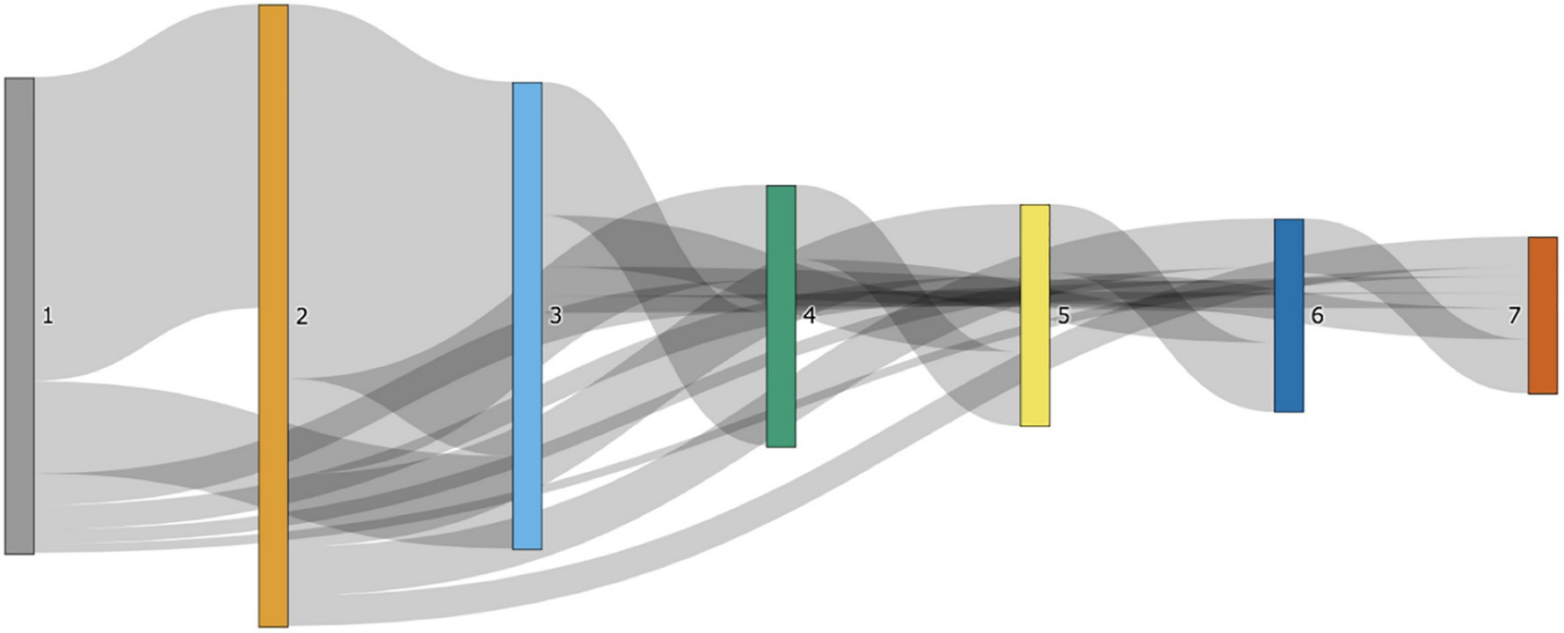
Flow of re-takers across seven iterations.

By inspecting enrollment data from all iterations, 16,570 learners (15.73% of the total) were identified as *re-takers*. Fig 1 presents the flow of re-takers across seven iterations. 75.26% (12,471) of all re-takers enrolled in consecutive iterations (i.e., flowing between adjacent iterations), while the rest learners re-took the MOOC after a longer time period (e.g., taking the 1st and 4th iterations of the MOOC). Table 2 presents a slightly different picture. It shows how the count of learning attempts in this MOOC was distributed. Among all learners, 84.27% of them learned only once (i.e., “one-timers”). Among the re-takers, 13,479 enrolled in the MOOC twice; 2,346 enrolled three times; and 745 enrolled four or more times.

**Table 2:** The distribution of repeated enrollment in the MOOC.

Count of attempts	Count of learners	% of all learners	% of re-takers
1	88,800	84.27%	–
2	13,479	12.79%	81.35%
3	2,346	2.23%	14.16%
4	546	0.52%	3.30%
5	144	0.14%	0.87%
6	41	0.04%	0.25%
7	14	0.01%	0.08%

Demographic information of re-takers and one-timers is presented in Table 3. Overall, re-takers were mostly female (65.69%), with a median age of 35. A higher proportion of re-takers were Grade 7 to college teachers, whereas one-timers had a higher proportion of college students. The majority of re-takers (72.57%) were at the *Experimentation and reassessment* stage of their careers as defined in [67], substantially more than one-timers percentagewise. All 34 Chinese provinces, autonomous regions, and municipalities were represented.

**Table 3:** Demographic characteristics of re-takers and one-timers.

Variable	Category	Re-takers	One-timers
Sex	Male	34.31%	39.00%
	Female	65.69%	61.00%
Affiliation	K-6 school teachers	15.72%	16.03%
	Grade 7-12 teachers	33.70%	29.06%
	University and college teachers	31.38%	29.36%
	College students	6.67%	12.14%
	Others	12.53%	13.41%
Age and career stage	≤ 22 Pre-career	3.47%	8.94%
	23-25 Career entry	5.41%	8.37%
	26-28 Stabilization	7.60%	8.73%
	29-47 Experimentation and reassessment	72.57%	63.23%
	≥ 48 Serenity and conservatism	10.95%	10.73%

### Data sources

To investigate the phenomenon of course re-taking and networked professional learning in this MOOC, we used both qualitative and quantitative data sources including course enrollment records, survey responses, click logs, learning performance data, and semi-structured interviews. Data sources included:

1. Enrollment data. MOOC re-takers were first identified using enrollment data from all offerings of this MOOC, by detecting learner IDs that appeared in multiple iterations.
2. Entry survey data. At the beginning of each course iteration, learners were invited to complete a survey comprising questions about their demographic backgrounds (e.g., region, age, educational background), learning motivations, and intended learning efforts in the course. The current study focused on the responses from the re-takers.
3. Learning performance data. This course was designed with a number of quizzes, assignments, discussion activities, and summative assessments. Course assignments involved drafting and critiquing lesson plans that incorporated the Flipped Classroom approach and were therefore relevant to most learners’ practical contexts. Learner performance on these assessments was collected for analysis.
4. Click logs recording learning behaviors. Click logs from the MOOC consisted of two types of event: *page views* and *video clicks*. Page views included information about the user, timestamp, and the URL of the corresponding page. The URL could be used to identify (a) the type of action (e.g., reading vs. creating a forum post), and (b) the related course item (e.g., a lecture video, a quiz). For video clicks, the log data showed information about the video that was viewed and the point at which the video was played, paused, etc. Because the MOOC provider was only able to provide click logs from courses offered after 2015, this study analyzed the click logs of the 4–7th course iterations, totalling 5 million user-generated events.
5. Semi-structured interviews. Nine MOOC re-takers participated in hour-long semi-structured phone interviews. They represented varied levels of learning engagement and performance. Following the sequential explanatory mixed-methods design [64], the aim of the interview was to construct a rich, qualitative picture of their learning intents, their access to PD in professional settings, and their experiences of learning with the MOOC. Two researchers conducted the interviews. They took notes and created various “in-vivo codes” during the interviews, and later transcribed the audio recordings for analysis.

### Data analyses

A wide range of data analyses were conducted to address the research questions. Survey and click logs were analyzed using statistical and data mining techniques to discern patterns (more details below). The interview data were coded by three researchers following the *grounded theory* approach [68] to generate codes and themes that could help explaining patterns identified from quantitative analyses. The qualitative coding comprised two main steps. First, researchers conducted open coding focusing on what re-takers did within and outside the MOOC. Based on in-vivo codes created during the interviews and results of the opening coding, researchers then conducted axial coding, leading to different categories and properties of teachers’ networked professional learning as well as rich information to describe them. To achieve reliability of coding, we first trained the coders in two separate meetings. They then coded one same interview transcription independently and discussed results to reach agreements. They then coded all nine interviews and conducted inter-rater reviews before finalizing the codes.

To answer RQ1, we conducted descriptive statistical analysis of learning intents using the entry survey data, by especially comparing re-takers with one-timers. We also examined patterns in course performance data and identified four groups of re-takers: re-takers who received high scores in their first and second enrollments (*high–high* scores), those who received a high score in their first enrollment and a low score in their second enrollment (*high–low* scores), those who received a low score in their first enrollment and a high score in their second enrollment (*low–high* scores), and those who received low scores in both enrollments (*low–low* scores). By examining the distribution of these four re-taker categories, we analyzed how the learning performance changed across two adjacent learning attempts of the MOOC and examined the extent to which course re-taking was related to the probable motivation of improving course performance. Based on the categorization, we then turned to qualitative codes of the interview data to examine the re-takers’ learning needs, motivations, and the extent to which these needs were met by the MOOC.

To answer RQ2, we examined re-takers’ behaviors within the MOOC by applying *frequent pattern mining* (FPM), a popular data mining technique [69, 70], to the MOOC click logs. The *arules* R package was adopted for this analysis [71]. In this analysis, we were interested in identifying frequent *itemsets*, each of which comprised a set of web links frequently accessed by a same learner during one MOOC iteration. By comparing frequent itemsets identified from different clusters of re-takers during different iterations, we would uncover behavioral patterns of re-takers when they learned within the MOOC. Qualitative analysis of interviews provided further insights into these patterns of re-takers’ learning behaviors in the MOOC.

When answering RQ3 related to the connection between re-takers’ MOOC learning and workplace experiences, we primarily drew on qualitative coding of interviews. The qualitative coding described in the first paragraph of this section uncovered themes regarding the ways re-takers connected the MOOC with their professional settings. In particular, the results of coding enabled us to answer RQ3 by extracting themes of teachers’ networked learning and constructing a detailed picture of each theme. These analyses, combined with previous quantitative analysis, would help us understand the extent to which MOOC re-takers were engaged in networked professional learning.

## Results

### What motivated teachers to re-take the MOOC?

**Fig 2:**
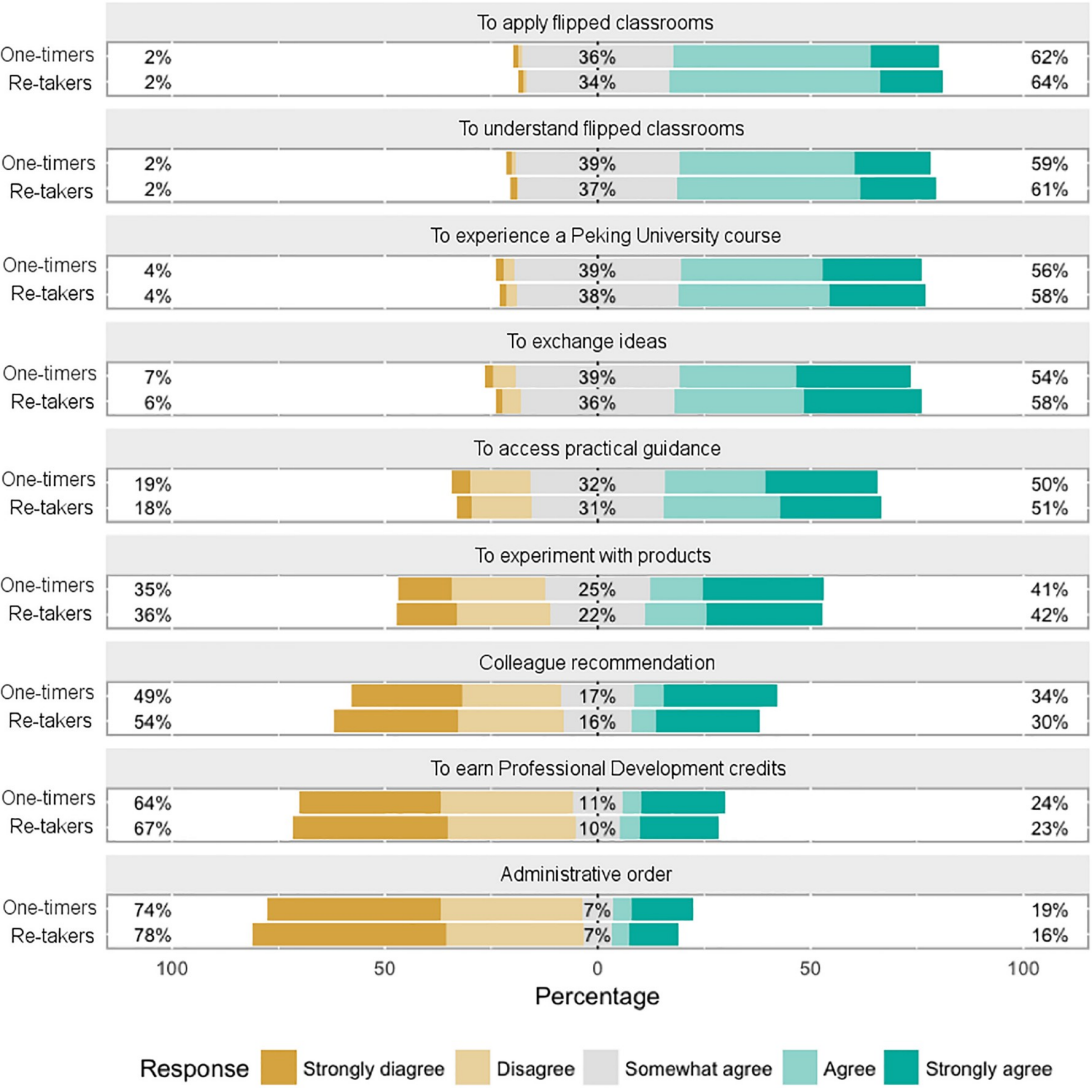
Learners’ motivations of taking the MOOC.

The entry survey of this MOOC included questions about learning motivations and intents. As demonstrated by Fig 2, the strongest motivations were related to applying and understanding the Flipped Classroom approach (more than 60% *Agree* or *Strongly Agree*), and less learners took the MOOC under administrative orders or to earn professional development credits (less than 20% *Agree* or *Strongly Agree* for both). Statistical analysis did not find any significant differences between re-takers and one-timers in terms of learning motivations.

However, a separate item in the survey revealed re-takers had a stronger intent to excel in this MOOC. A chi-square test of the dependence between this particular intent and the type of learner was significant, *χ*^2^(5) = 18.08, *p* < .01. More re-takers intended to earn the excellence certificate than one-time learners, and fewer of them intended to merely browse course content.

In terms of the learning performance, in the first attempt both re-takers and one-timers showed a highly similar U-shaped curve distribution of their grades. For the first learning attempt, re-takers achieved a higher median score (*Md* = 47.40) than one-time learners (*Md* = 21.98). The highest score achieved by a re-taker was expected to be significantly higher (*Md* = 67.91) than the score of a one-time learner (*Md* = 21.98; *z* = 7.31 × 107, *p* < .001).

So a follow-up question was: Did re-takers re-take the MOOC to improve their course performance? We examined the change of scores of re-takers across different iterations of the MOOC. Results showed that 44.76% of re-takers attained zero point across multiple attempts, and another 24.27% did not attain a passing grade from multiple attempts. Among those who received a passing grade (30.97%), 31.87% ceased re-enrollment when eventually obtaining the certificate after multiple attempts; in contrast, the other 68.13% continued to take the MOOC after attaining a passing grade, including a notable 43.07% who achieved an *Excellent* grade (i.e., greater than 80%) but still returned to the MOOC. Notably, while some re-takers re-enrolled to obtain the certificate, a greater proportion of re-takers returned to the MOOC even after passing the course. Apparently, many teachers formed a strong connection with the MOOC and they did not solely use the connection to improve course grades.

**Fig 3:**
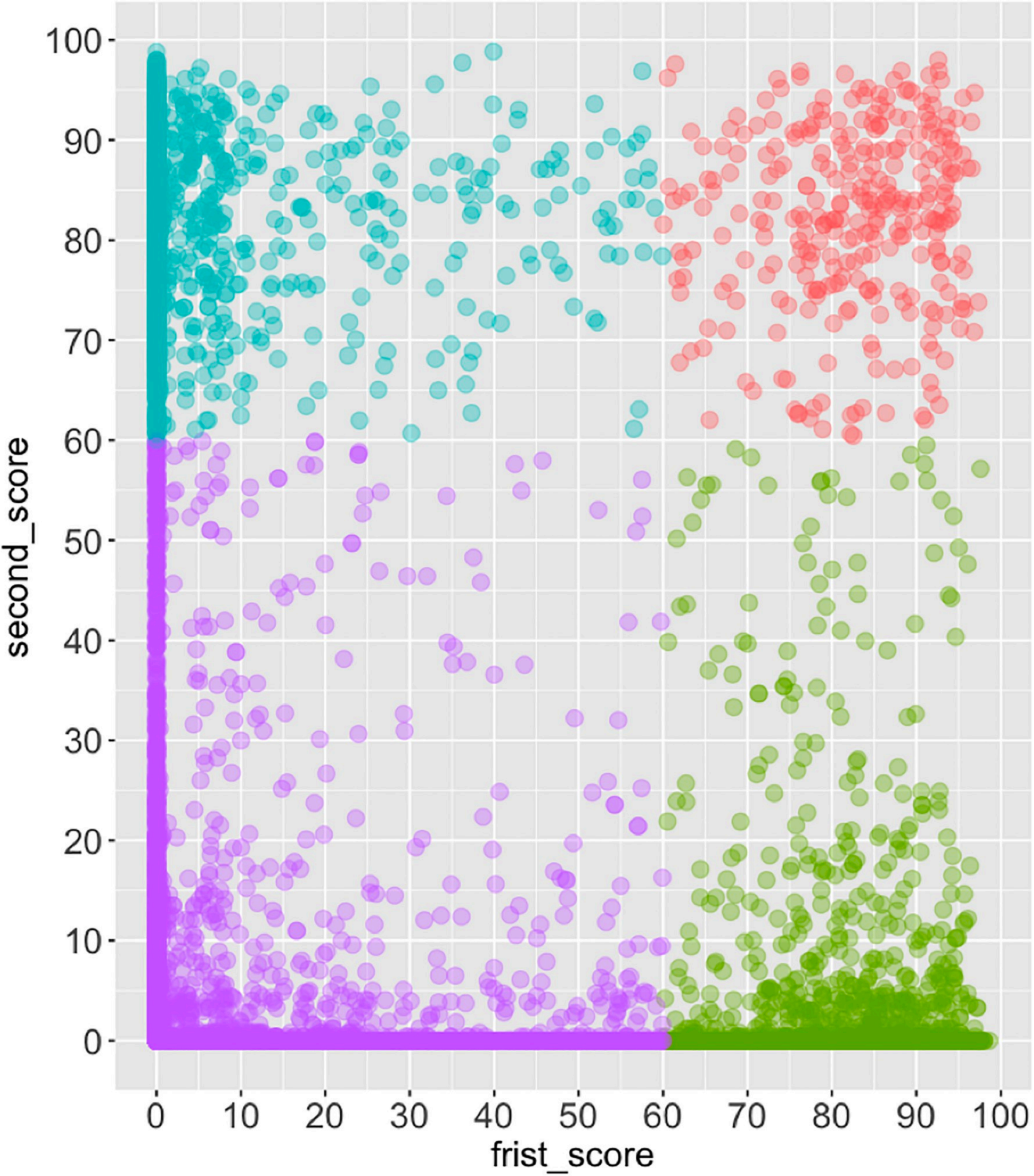
Scatter chart for the grades of re-takers. Two adjacent terms are plotted.

Fig 3 demonstrates the scores of re-takers from two adjacent learning attempts. In this figure, two adjacent scores are respectively represented by the x- and y-axes. The figure shows that a large number of learners achieved relatively low scores in both learning attempts (represented in purple; Quadrant III). Learners in the upper-left quadrant (Quadrant II, blue) obtained relatively low scores from their first attempt, but achieved higher scores, and in many cases above 60%, the second time. Learners in the lower-right quadrant (Quadrant IV, green) attained scores above 60% on the first attempt but low scores on the second attempt. A fourth group of learners, represented by red dots in the upper-right quadrant (Quadrant I), achieved relatively high scores in both attempts, with some achieving even excellent grades (more than 80%).

Interviews with MOOC re-takers provided possible explanations of the quantitative findings. For some re-takers, the MOOC certification was relatively less important in comparison with improving teaching practice. For example, Mr. C expressed a disinterest in gaining a certificate from this MOOC even though he was proud of receiving the certificate regardless of his heavy workload. He explained that the certificate he received “felt more like a record of his own investment in learning.”

The same sentiment was shared by Ms. W:

This certificate has little effect on my career. The province does not recognize any credits of continuing education received through MOOCs. But I think that I have to push myself to obtain a certificate after taking such a great course. This goal urged me to stick to it till the very end.

Another example was Mr. L, who recommended the course to his colleagues because the course could help alleviating difficulties in teaching. His colleagues were not drawn to the MOOC by the certificate either. As Mr. L put it:

My colleague did not care about the certificate at all. He did not mean to get the certificate as long as he could improve his teaching.

Of course, these viewpoints held by some participants did not mean that receiving the certificate was not important at all. For some other participants, they were initially “hooked” by the MOOC because of external incentives while they gradually developed intrinsic motivation as learning progressed. For instance, Mr. Z was first introduced to this MOOC by his principal during a school meeting.

The principal encouraged teachers to keep refreshing their teaching strategies through MOOCs during the summer vacation. He also promised to reimburse the certification fees and provide extra awards to those who could complete the course… So initially I was motivated by the rewards… then I started taking these courses and became absolutely fascinated by its substantive content. Thereafter I cared much less about certificates or those rewards, all because the course content was so good.

Ms. K did not rely on incentives or rewards to enter the course, but she felt compelled to complete the course and to get the certificate. According to her, such a sense of compulsion served her well.

Because I wanted to get a certificate, I was very conscientious about my study, and the result was much better than just going through it casually.

Indeed, the original motivation for some re-takers was to receive the certificate but the learning motivation shifted as learning progressed. For some re-takers, learning evolved from a noun to a verb, from an end product that should be certified to a process that involves time spent on MOOC modules and efforts made to change their own teaching.

### What did re-takers do within the MOOC?

To investigate learning behaviors within the MOOC, frequent pattern mining (FPM) was applied to the click logs from the 4-7th course iterations (given click logs from earlier iterations were not accessible). In preparation for FPM, we divided re-takers from the 4-7th iterations into four quadrants according to the previous analysis of learner performance across multiple attempts (see Fig 3). By doing so, we were able to examine the re-takers in each quadrant by mining their behavioral patterns of each learning attempt. As shown in Table 4, learners were not evenly distributed in four quadrants. In particular, comparatively fewer learners (*n* = 16) from these course iterations received course certificates in both attempts, while a majority of learners failed to pass the MOOC in both attempts (*n* = 2,864). In Table 4, the total numbers of recorded click events are also reported, respectively for each attempt by re-takers from each quadrant. It is evident that the average number of click events was predictive of course performance. For example, learners from Quadrants II and IV generated more clicks in their successful attempts; the average number of clicks in Quadrant III learners was low and they were unsuccessful in passing the course in both attempts.

**Table 4:** Results of frequent pattern mining of click streams of re-takers.

Learner quadrant	Attempt	# of events	Frequent itemsets (top 4, sorted by *lift*)
Quadrant II (n = 342)	1	9,441	{1106,1109,1110,1212,1214,1215} {1106,1108,1109,1212,1214,1215} {1106,1108,1109,1110,1212,1214} {201_chat,202_chat,2105,2207}
	2	52,809	{5207,5208,5209,5210,5211,5212,5213,5318,5319} {2209,502_chat,5207,5208,5209,5210,5211,5212,5213,5318} {3207,3209,3211,3212,3213,5207,5208,5209,5210,5211} {501_chat,502_chat,5207,5208,5209,5210,5211,5212,5213,5318}
Quadrant III (n = 2,864)	1	43,344	{1212,1214,1215,1317,1319,1321,1323} {1108,1109,1110,1212,1214,1215,1317,1319} {1106,1108,1109,1110,1212,1214,1215,1319} {1106,1108,1109,1110,1212,1214,1215,1317}
	2	21,178	{1106,1108,1109,1110,1212,1214,1215,1317} {101_chat,1106,1108,1109,1110,1212,1214,1215} {1108,1109,1110,1212,1214,1215,1317} {1106,1109,1110,1212,1214,1215,1317}
Quadrant IV (n = 233)	1	46,508	{202_chat,2209,2211,2213,2215,2217,2322,2324,2326,2329} {202_chat,2207,2209,2213,2215,2217,2322,2324,2326,2329} {101_chat,201_chat,207_chat,208_chat,2322,2324,2326,2329,2431,2433} {2105,2207,2209,2211,2213,2215,2217,2322,2324,2326}
	2	768	{3206,3207,3209,3211,3212,3213,3214} {3207,3209,3211,3212,3213,3214} {3206,3207,3209,3211,3212,3213} {3206,3209,3211,3212,3213,3214}

As mentioned in the *Data sources* section, clickstream data from only the 4-7th course iterations were available for analysis, leading to a mismatch between numbers of learners between Fig 3 and this table.

**Fig 4:**
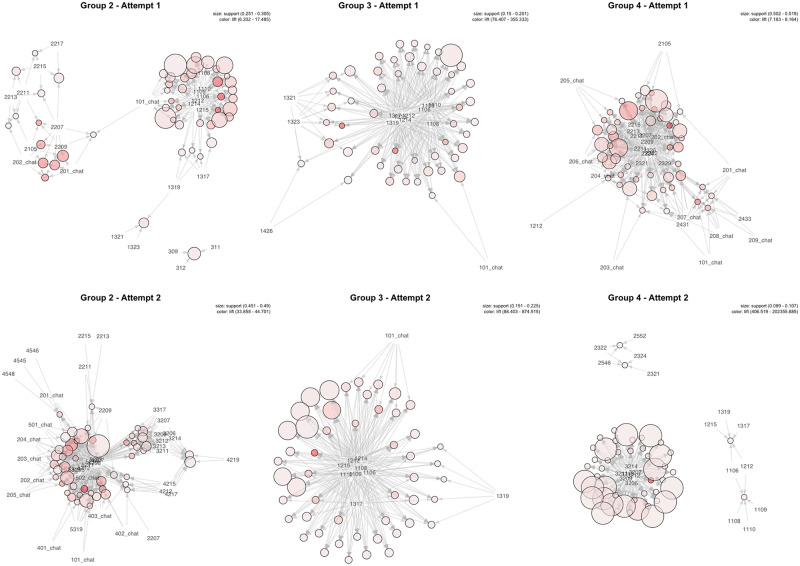
Visualizations of frequent itemsets of student groups in two attempts. In each graph, each frequent itemset is visualized as a circle and its contributing items (in codes) pointing towards identified frequent itemsets. The size of a circle indicates its *support*, and the color represents *lift*. See [71] for more information about these measures.

Next, FPM was applied to identify clusters of course resources each quadrant of perpetual learners gravitated towards. For Quadrant I, because this quadrant had relatively few learners, the mining of frequent itemsets was less meaningful and was not included in the results. In Table 4, the top four frequent itemsets, judged by *lift* (a measure of a frequent itemset’s interestingness [71]), are listed for each attempt by learners from each quadrant. In this list, each web URL is coded based on its position in the course. For example, item 3207 is the 7th web page of the MOOC’s Module 3.2. Results of frequent itemsets are also illustrated in Fig 4, with each frequent itemset visualized as a circle and its contributing items (in codes) pointing towards identified frequent itemsets.

The top frequent itemsets revealed the following key patterns:

- Quadrant II. Frequent itemsets from the first attempt (unsuccessful) were mostly focused on the first two modules, whereas those from the second attempt (successful) were concentrated in latter modules (especially Module 5).
- **Quadrant III**: Frequent itemsets from both attempts were concentrated in the first module. Learners did not venture far in this course in either attempt. One notable difference between two attempts was that course item 101_chat appeared in multiple frequent itemsets of the second attempt (see Table 4 and Fig 4), indicating more discussion behaviors in learners’ second attempt even though they still failed to pass the course.
- **Quadrant IV**: In the first attempt, items from the 2nd module, especially discussion activities (e.g., 202_chat) were well represented. In the second attempt, learners’ behaviors were focused on Module 3 that was focused on software tools for flipping the classroom. Notably, many learners in this quadrant, who were successful in their first attempt, were probably integrating the Flipped Classroom approach in their teaching and came back to the MOOC to refresh their technical skills.

Qualitative analysis of interviews provided further insights into the re-takers’ learning experiences within the MOOC. Frequent pattern mining indicated some learners came back to refresh technical skills pertinent to the Flipped Classroom approach. The openness of the MOOC enabled learners to re-take the course multiple times, return to the course if needed, and refresh specific topics as they work to solve problems in their teaching practice. Teacher interviews confirmed the existence of this pattern. For example, Ms. Z, from Quadrant IV, shared an episode of a teaching demonstration she attended. She witnessed teachers and researchers debating the need to plan a lesson down to the most minute detail. She realized this debate was connected to “the golden rule” discussed in MOOC, so she went home, opened the MOOC, found the lecture video, and re-watched it with the debate in her mind.

Mr. Z compared this type of just-in-time learning with traditional time-out for learning. For him, teacher training in the past were just like a meeting. “There are no goals. We just listen and take notes.” He noted that “it is difficult to sustain professional learning without a clear purpose or without a need to solve specific problems.” He believed the MOOC offered opportunities for him to continually address problems in teaching, reflecting important characteristics of networked professional learning [47].

For Mr. P, such networked professional learning with MOOCs prompted him to believe that today’s teachers need to research one’s own teaching, actively resolve encountered problems in teaching, and take initiatives to improve one’s teaching practice. This belief is congruent with the broader conception of “worker as learner” [42] and emphasizes the active development of knowledge-in-use [45] in the teaching profession. Such a desire to continually improve one’s teaching was concurred by Mr. Z:

I just want to make a change after taking this course. I keep in mind that to stay unchanged is out of the question. We must retain what we have done well in the past and meanwhile improve what we have not done so well. At this time, the biggest inspiration I got from taking this MOOC was about student evaluation. It inspired me to design a point system for my Chinese literature course.

In traditional one-shot workshops or lectures, the learning and work environments of the teacher are often isolated, making it difficult to dynamically connect teaching practice and professional learning. In contrast, these re-takers maintained a connection with the MOOC and harnessed the connection to continually improve their work by fusing in-course learning with their professional practice.

### What did re-takers do with the MOOC in their professional settings?

Besides re-entering the MOOC, some re-takers took the initiative to facilitate learning groups in their organizations or communities. Mr. L, the director of a university teaching center, took this MOOC himself and immediately recognized its value for an ongoing teaching reform at his university. He recruited a group of teachers to take the MOOC, and he hosted offline meetings for further discussions.

I organized group study as well as offline seminars for the teachers at my university. The seminars were related but not limited to the MOOC. Everyone would have opportunities to share their journeys of professional learning and recommend new MOOCs and new resources to others… It created a great atmosphere for the school’s teaching and teacher development.

The MOOC also allow K-12 teachers to connect and form groups with peers from other schools. As Ms. Y shared,

We met initially through the MOOC’s discussion forum. We realized that several local high schools were not far away. We were all willing to flip our classrooms, so we would observe and analyze each other’s class, discuss our thoughts together, and even dine together sometimes. And now we are planning to apply for funding to launch a few research projects together.

However, not all teachers were able to develop a supporting structure for their professional learning with the MOOC. Many teachers found it difficult to get support from their schools and colleagues. For instance, Mr. P, who was in charge of improving the teaching practice of his school, reported his colleagues’ disinterest in this MOOC. As a result, he had to use his executive power to put more teachers into the MOOC even though he was worried this strategy might be counterproductive. Another teacher, Mr. Z had a difficult time seeking endorsement from his school principal for his visits to other schools to extend his learning. “The leaders did not approve…” as Mr. Z shared, “They didn’t really want to send me out for further study. They found the procedures troublesome and they were unwilling to cover the expenses.”

For some teachers who could not easily find a supporting environment locally, they resorted to the MOOC and built online communities of practice around the MOOC. Many teachers created informal groups on social media (such as WeChat) to stay connected. When encountering problems in teaching, Mr. K “preferred to resort to the [social media] group for help” or to find “like-minded friends to talk with.” In his situation, he had been attempting to flip his classroom but the other teachers in his school have not heard about the concept. He felt “a bit lonely, because people around [me] did not understand [my] difficulties.” So he formed an online group to address difficulties with like-minded peers from other regions. Mr. K said,

With this group, I could throw any problems for extended conversations. I was most delighted to see I was not the only one facing these challenges. It made me willing to positively respond to their challenges.

Having shared interests or shared practical problems motivated the formation of these informal groups outside of the MOOC. As some teacher-learners were traversing multiple MOOCs, they realized the importance of having shared understandings and vocabularies for productive conversations. As Ms. L mentioned,

People’s knowledge backgrounds were drastically different. Sometimes the discussion was highly inefficient because they failed to reach a consensus on the most basic concepts… Everybody had their own interpretations, leading to a weak basis for meaningful discussions.

In contrast, for those teachers who have taken the same MOOC before, meaningful discussions were more likely to happen. As Mr. Z put it,

Because everyone had taken the MOOC taught by Mr. W, that MOOC helped us establish a shared understanding. At least there was no need to waste any time on discussing various basic issues, which saved time for deeper dialogues.

Since the MOOC mentioned by this participant was offered by another professor, it appeared these teachers extended their connections across multiple MOOCs. Having a stable community of practice with shared knowledge, interests, and practical concerns was helpful for them to engage in deeper conversations that were otherwise impossible in their local schools or with less familiar peers in the MOOC.

Over time, some re-takers chose to stay connected not only because of their own learning needs but also for their self-image of “helpers” and “leaders” in the MOOC learning community. As Ms. L said,

In fact, my interpretation of the MOOC is that it is an ecology. … For example, I will continue to register for our MOOCs after completion. I am an “old” or experienced learner. In comparison to new learners of the MOOC, I can contribute to this ecology, encourage others, and answer questions. As an experienced learner, I see myself as an enthusiastic supporter. I do get recognized for my effort and have a sense of accomplishment and belonging.

## Discussion

### Addressing research questions

MOOCs are popularly used to support teacher professional development at scale [72, 73]. Given the changing landscape of professional learning, e.g., from timing-out to learn to just-in-time learning and work-as-learning, MOOCs offer a compelling scenario for teachers to engage in networked professional learning that connects learning opportunities with their teaching practice. This study was conceived to investigate a unique phenomenon—MOOC re-taking by in-service teachers who kept returning to the same MOOC that was offered for multiple occasions. By doing so, we probed the potential of using MOOCs to support networked professional learning as an alternative approach to teacher PD in the information-rich, networked society. We asked three major research questions about MOOC re-takers regarding their diverse learning motivations, their behaviors within the MOOC, and the connections they forged between the MOOC and their professional settings.

First of all, more than 15% of all learners took the MOOC for multiple times and fell under our definition of re-takers. They were mostly female and predominantly at the Experimentation and Reassessment stage of their teaching careers. Compared to one-time learners, the re-takers revealed a stronger intent to excel in this MOOC even though they were similarly motivated by the opportunity to apply the MOOC content. In terms of course performance, the highest score achieved by a re-taker was higher than that of a one-timer. However, re-takers did not necessarily re-take the course to better their course performance. In fact, a majority of the re-takers did not earn a passing grade. While some re-takers successfully passed the course after multiple attempts, some others returned the MOOC after earning a desirable grade and then performed poorly in the re-attempts.

Frequent pattern mining following different performance trajectories revealed that different categories of learners focused on different parts of the MOOC during their attempts. Notably, learners who failed first and passed later were able to venture further in the course. Learners who completed the MOOC on the first attempt and failed the second most probably came back to refresh technical skills involved in flipping the classroom.

In-depth interviews with select participants revealed authentic knowledge needs that motivated them to return to this MOOC. Even though some of them were attracted to the MOOC by external incentives, they discovered the value of professional learning facilitated by this MOOC. Many of them had a desire to continually improve their teaching practice and some demonstrated strong reflexive thinking on the teaching profession [74, 75]. Some learners took initiatives to create support groups and communities to help them “stick around” and collaboratively solve practical problems. By sustaining the connection with this MOOC, some teachers created new connections with colleagues within their organizations and on social media to support each other’s professional learning [37, 47]. The nuanced picture of what they were doing with the MOOC opened up the space for envisioning a more holistic supporting structure for the professional development of in-service teachers, a structure that enables stronger fusion between work and learning, facilitates just-in-time learning, and empowers them to act as change agents in their education systems.

In general, our analysis showed that MOOCs could potentially serve diverse learning needs of course re-takers. More importantly, we observed the significance of sustaining the connection between teachers and the MOOC, as well as the value of creating various connections around this MOOC—local or online—for networked learning. While researchers argue that a MOOC can act as a key center for networked learning activities of “invisible leaners” who appear to be non-active and disengaged [76], we found “re-takers” harnessed the sustained access to a same MOOC to support their professional learning. Our findings provided glimpses into the richness of networked professional learning by in-service teachers, the ways in which they fuse work and MOOC learning, their diversified attitudes towards learning certification, and the connections they created between the MOOC and their organizational contexts. These results revealed the prospect of supporting networked professional learning of in-service teachers with MOOCs.

### Limitations

Findings from this study need to be interpreted with a few limitations in mind. First, the research team had constrained access to certain data sources and this reality limited the extent to which we were able to align different data analyses. For example, the Chinese MOOC provider we worked with could only provide access to system logs of the later iterations of this MOOC. As a result, we were unable to apply frequent sequence mining to participants from earlier iterations. Second, the percentage of re-takers who filled out the MOOC entry survey multiple times was very low. This limitation did not allow us to compare within-subject change with learning motivation across different learning attempts. Hence, findings from this study can only be generalized with great caution and important questions remain to be answered in future studies.

### Practical implications and future directions

Findings from this study have practical implications for teacher professional development, especially for regions where teachers have limited access to quality professional development. First, different trajectories of course performance and different behavioral patterns of in-MOOC learning implied the need to better understand teachers taking these MOOCs. The illuminated patterns of re-enrollment by in-service teachers indicated a strong desire to stay connected with the MOOC. Traditionally, MOOCs are catered to one-time learners without much consideration of various learning intents and goals held by re-takers. Future MOOCs, especially those designed for in-service teachers and other working professionals, could tailor learning design to better serve networked learners who may stick around for better grades, course content, and/or peer connections. In addition, the study revealed substantial informal organizing work done by learning professionals to create local learning groups and online communities of practice. It is thus desirable for MOOCs created for teachers to pay more attention to community-based learning, so as to catalyze encounters among teachers and to facilitate network formation and knowledge building around authentic problems [51, 52].

The strong connection between in-service teachers’ learning in the MOOC and their teaching practice is also worth attending to. Traditional teacher training seminars tend to be disconnected from real-world teaching, resulting in difficulties in applying new knowledge to teaching practice. In this study, some teachers who continued to return to this MOOC forged strong connections between the MOOC content and their teaching experiences. Hence, MOOCs for teacher learning can be designed to encourage discussions of real-world teaching problems and sustain teacher problem-solving. Even though MOOCs can serve as a lever for teacher professional development at scale, based on the study we are even more intrigued by the *quality* of teacher learning MOOCs can promote by making the utmost effort to foster connections to empower teacher learning across contexts and for extended periods of time.

We also recognize the importance of involving local administrators (e.g., education bureaus, school principals) to create a comprehensive supporting structure for networked professional learning for teachers. As shared by some teachers, they were strongly encouraged by their principals to take the MOOC and later recognized its value for their professional growth. In contrast, some teachers felt lonely and were poorly supported by their schools and colleagues. Unlike MOOCs on topics less connected with a profession, the uptake of MOOCs designed for teachers can be promoted in close collaboration with existing educational institutions. It is therefore important to explore synergies with educational administrators to further tap into the potential of MOOCs for teacher professional development.

There are several promising future directions to deepen this work. First, while this study was focused on re-takers of a single MOOC, it would be interesting to examine learners who take many different MOOCs (cf. re-takers who take a single MOOC multiple times). This unique sub-population of MOOC learners has been documented in [12] and in the present study. Investigating their learning experiences could help us understand the extent to which learners mobilize peer connections to support learning across MOOCs. Second, much conceptual work needs to be done on the notion of professional networked learning which is still nascent for teacher PD [77]. Finally, as suggested by both networked learning and our research findings, it would be desirable to pivot on networks and connections when designing future teacher learning MOOCs.
